# The Relation between Epicardial Fat Thickness and Prognostic Risk Scores

**DOI:** 10.5935/abc.20160185

**Published:** 2016-12

**Authors:** Sevket Balta, Cengiz Ozturk, Turgay Celik

**Affiliations:** Department of Cardiology, Gulhane Medical Academy, Ankara, Turkey

**Keywords:** Acute Coronary Syndrome / complications, Adipose Tissue, Pericardium, Echocardiography, Score GRACE

**Dear Editor,**

We read the article "The Relationship between GRACE Score and Epicardial Fat Thickness(EFT) in non-STEMI Patients" by Gul et al.^1^ The authors concluded that end-systolic and end-diastolic EFTs were found to be increased in the higher GRACE group.

Several risk scores including have been evaluated so as to identify high/low risk of mortality or complications in patients with STEMI. These methods have been extensively evaluated for the follow-up of short or long term events.^2^ However, some studies showed that the GRACE risk score had low predictive accuracy for mortality. On the other hand, other studies showed that the GRACE risk score had the highest prognostic accuracy for long-term mortality.^2^ For this challenge, these risk scores may not give accurate information about high/low risk of mortality or complications in patients with STEMI. So, several risk scores should be evaluated when assessing the relation between any of the risk scores and high/low risk of mortality or complications in patients with STEMI.

Furthermore, epicardial tissue as a endocrine organ releases a lot of markers that may be related to oxidative stress, inflammation, endothelial dysfunction and atherosclerosis. Epicardial adiposity can also be associated with cardiovascular mobidity and mortality.^3^ Furthermore, EFT is also associated with hyperlipidemia, obesity, diabetes, hypertension, smoking, and carotid atherosclerosis, as well as with cardiovascular risk factors like metabolic syndrome, nonalcoholic fatty liver disease, and chronic kidney disease.^4^ Moreover, some endocrin tissue dysfunction like thyroid issue is associated with EFT. Furthermore, some inflammatory diseases like psoriasis have higher EFT values and EFT may be a possible marker of endothelial dysfunction and increased cardiovascular risk in patients with psoriasis^5^

GRACE risk score may give about mortality or complications in patients with STEMI, but both use the risk scores or inflammatory markers. We believe that these findings will evaluate further studies about EFT and GRACE risk score in STEMI patients.
